# Sibling relationships of female adolescents with nonsuicidal self-injury disorder in comparison to a clinical and a nonclinical control group

**DOI:** 10.1186/s13034-019-0275-2

**Published:** 2019-03-15

**Authors:** Taru Tschan, Janine Lüdtke, Marc Schmid, Tina In-Albon

**Affiliations:** 1 Clinical Child and Adolescent Psychology, University of Koblenz-Landau, Ostbahnstraße 12, 76829 Landau, Germany; 2 Department of Child and Adolescent Psychiatry, University of Basel, 4056 Basel, Switzerland

**Keywords:** Nonsuicidal self-injury, Sibling relationship, Sibling agreement, Family

## Abstract

**Background:**

Adolescents’ nonsuicidal self-injury (NSSI) leads to distress that affects the whole family system, and siblings are reported to suffer from disrupted family communication and functioning. So far, no studies have examined the quality of relationships between adolescents with NSSI and their siblings. The aim of the present study was to examine the sibling relationship quality of adolescents with NSSI, adolescents with other mental disorders without NSSI (clinical controls, CC), and adolescents without current or past experience of mental disorders (nonclinical controls, NC).

**Methods:**

139 female adolescents aged 13–20 years (mean age = 16.18 years, *SD* = 1.62, NSSI: *n* = 56, CC: *n* = 33, NC: *n* = 50) and 73 siblings aged 10–28 years (mean age = 16.88 years, *SD* = 4.02, 60.3% female) participated. Self-report measures were used to assess psychopathology and sibling relationship quality.

**Results:**

Siblings reported a wide range of negative emotional and familial consequences, such as feeling left alone with their sister’s issues or a distressing family situation, as a result of their sister’s NSSI. Siblings of adolescents with NSSI experienced significantly more coercion in the relationship with their sister compared to CC (*d* = 1.08) and NC (*d* = 0.67) siblings, indicating an imbalance of dominance and control in their relationship. Further, adolescents with NSSI reported significantly less warmth and empathy in the sibling relationship and higher rivalry scores between their siblings and themselves than NC adolescents, suggesting higher levels of parental favoritism among parents of adolescents with NSSI compared to NC parents (*d* = 0.93). Among siblings of adolescents with NSSI, high levels of warmth, conflict, and empathy were significantly associated with internalizing problems. For adolescents with NSSI a significant association was found between internalizing problems and coercion and externalizing problems and similarity.

**Conclusions:**

Given the negative impact of NSSI on siblings’ emotional well-being and family life, efforts should be made to offer siblings psychoeducation and support to help them cope with the emotional and familial consequences of their sister’s NSSI. Given adequate support, siblings can in turn be a source of emotional support for their sister.

## Introduction

Nonsuicidal self-injury (NSSI) is a highly prevalent behavior among adolescents and associated with various mental health problems and suicidality [1–3]. NSSI is defined as the repetitive, deliberate, direct, and socially unaccepted destruction or alteration of one’s own body tissue without the intent to die [4]. Pooled international lifetime prevalence rates among adolescents (including single acts of NSSI) are around 17% [5], with 6.7% [6] reporting repetitive NSSI according to *DSM*-*5* criteria [4]. Females are more likely to report a history of NSSI than men, particularly in clinical samples [7].

Previous research has emphasized the role of maladaptive family functioning, such as emotional invalidation and lack of family support, as crucial proximal risk factors for the development of NSSI [8–13]. Contrary, family support and positive family functioning were found to predict the cessation of NSSI [10, 11, 14]. Similarly, a review on psychosocial treatment for self-injurious thoughts and behaviors concluded that a crucial part of efficacious interventions is improving familial relationships [15]. However, research on familial relationships in the context of adolescent NSSI has so far focused primarily on parent–child relationships, while remarkably little is known about sibling relationship quality. The sibling relationship is life’s longest lasting and one of the most important relationships, as children spend more time with their siblings than with their parents [16]. Sibling relationships encompass positive (e.g., warmth, intimacy, empathy) and negative (e.g., conflict, rivalry) features and can have a major impact on sibling’s lives and wellbeing (see [17] for a review). Social or observational learning are mechanisms to describe generalization of negative behaviors among siblings, such as hostile behavior [18].

A meta-analysis found that sibling warmth was significantly associated with less internalizing and externalizing problem behavior in children and adolescents [16]. Within positive sibling relationships, children and adolescents may learn favorable strategies to manage and regulate their emotions, leading to a lower risk of developing symptoms of depression, anxiety and aggression. On the contrary, sibling conflict was significantly related to more internalizing and externalizing problems [16]. Frequent fighting among siblings or observing a siblings hostile behavior might lead to generalization of negative behaviors to other contexts via social learning mechanisms [18]. Noteworthy, the association between internalizing and externalizing problems was stronger for sibling conflict than sibling warmth.

Furthermore, there is some evidence that children and adolescents with mental disorders have poorer sibling relationships compared to nonclinical individuals. Sibling relationships of children with attention deficit hyperactivity disorder (ADHD) are characterized by higher conflict but equal levels of warmth compared to children without ADHD [19]. Noteworthy, the authors suggest that comorbid internalizing and externalizing symptoms might be more powerful predictors of sibling warmth and conflict than ADHD per se. Moreover, poor sibling relationships in childhood and adolescence were found to predict the occurrence of major depression 30 years later [20]. Surprisingly, most research on sibling relationship quality and psychopathology include low-risk community samples [16], while there is a lack of research on sibling relationships of children and adolescents with clinically significant mental health issues including NSSI [17].

Adolescent NSSI behavior appears to impact the whole family system, leading to difficulties in parent–child relationships and disrupting family communication, family dynamics, and family functioning [21, 22]. Interview studies of parents’ reactions to their children’s NSSI behavior suggest that parents commonly have feelings of distress, insecurity, anxiety, guilt, and helplessness [21, 22]. Because parental time, energy, and attention is focused on the child with self-injuring behavior, parents express worries about an imbalance in parental involvement between siblings, particularly neglecting their other children [22–25]. Adolescents’ NSSI behavior and the distress it causes in the family likely affect siblings, especially if they are of a similar age, as these siblings, too, are trying to navigate through adolescence or young adulthood [22]. According to parents, siblings’ reactions to the NSSI behavior include a wide range of feelings such as anger, resentment, frustration, stress, simultaneous empathy and irritation, responsibility, worries about stigma at school, and often help and support [22]. Furthermore, some siblings have indicated feeling anxious about triggering an episode of self-injury with their own behavior [22]. To date, studies reporting data on siblings of adolescents with NSSI rely on parental reports, while no studies exist that assess sibling self-report with respect to their reactions to NSSI or sibling relationship quality.

It has been well documented that interpersonal conflicts often serve as triggers for engaging in NSSI [12, 26]. Adolescents with NSSI frequently report negative peer experiences such as peer victimization, which can significantly increase the risk of future NSSI [27]. Notably, the source of victimization may also be in the family; a longitudinal study [28] suggested that sibling bullying in early adolescence is significantly associated with NSSI behavior at age 18. Identifying risk factors for NSSI within the family might help researchers and clinicians better understand the familial mechanisms that are involved in NSSI and enable them to develop treatment modalities that include the improvement of familial relationships to save and improve the mental health of all family members.

The aim of the current study was threefold. First, we aimed to shed light on how siblings of female adolescents with NSSI feel about and evaluate their sister’s NSSI. Second, we wanted to investigate sibling relationship quality rated separately by adolescents with NSSI and a sibling. Previous research has indicated discrepant perspectives on family functioning and parenting behavior between adolescents with NSSI and their parents, with adolescents reporting poorer outcomes than parents [12, 29, 30]. Thus, we further aimed to examine the concordance between adolescent and sibling self-reported sibling relationship quality. Third, we wanted to explore the association between sibling relationship quality and psychopathology for adolescents with NSSI and their siblings, respectively. Specifically, we aimed to answer the following questions:

1. How do siblings react to their sister’s NSSI?
2. Do adolescents with NSSI differ from adolescents without NSSI (clinical and nonclinical controls) and from their siblings with respect to sibling relationship quality?
3. To what extent do adolescents and their siblings agree in their reports of relationship quality?
4. Is the sibling relationship quality associated with psychopathology in the NSSI/CC group?

## Methods

### Participants

#### Adolescents

The study included 139 female adolescents, aged 13–20 years (*M *= 16.18 years*, SD *= 1.62) that were consecutively recruited from different inpatient child and adolescent psychiatric units and schools in Switzerland and Germany. The sample comprised of 56 adolescents with NSSI disorder, 33 adolescents with other mental disorders without NSSI (clinical controls, CC), and 50 adolescents without current or past experience of mental disorders (nonclinical controls, NC). Participants were similar with respect to age, Welch’s *F* (2, 74.24) = 0.52. The most frequent mental disorders according to *DSM*-*IV*-*TR* of the NSSI group were depressive disorders (76%), anxiety disorders (48.2%), disruptive behavior disorders (22.2%), borderline personality disorder (18.5%), and eating disorders (18.5%). The CC group most frequently reported anxiety disorders (51.5%) and depressive disorders (45.4%), followed by eating disorders (24.2%) and disruptive behavior disorders (12.1%).

#### Siblings

Seventy-three siblings aged 10–28 years (*M *= 16.88 years, *SD *= 4.02; 60.3% female) participated in the study. We included only one sibling per adolescent, mainly the one closest in age. Overall, 27 brothers participated (NSSI = 12, CC = 1, NC = 14). Groups of siblings (NSSI = 21, CC = 11, and NC = 41) were similar with respect to age, Welch’s *F*(2, 20.79) = 0.72. A minority of siblings in the NSSI group (14.3%; 2 sisters, 1 brother) had had their own experiences with NSSI.

### Measures

To examine the adolescents’ current or past *DSM*-*IV*-*TR* diagnoses for Axis I disorders, we conducted a clinical structured interview. The Diagnostic Interview for Mental Disorders in Children and Adolescents (Kinder-DIPS) [31] assesses the most frequent mental disorders in childhood and adolescence. Questions for substance use disorders were included from the adult DIPS [32]. The Kinder-DIPS has good validity and reliability for Axis I disorders (child version, κ= 0.48–0.88) [33]. NSSI disorder was assessed according to the *DSM*-*5* research criteria, with questions reformulated as criteria. Interrater reliability estimates for the diagnosis of NSSI were very good (κ= 0.90). Before conducting the interviews all interviewers received an intensive standardized training.

Adolescents were administered the Structured Clinical Interview for *DSM*-*IV* Axis II disorders (SCID-II) [34], to assess for personality disorders. The SCID-II has been found to be suitable for use among adolescents [35]. Interrater reliability for borderline personality disorder in our sample was very good (κ= 1.00).

The Youth Self-Report (YSR) [36, 37] was used to assess a broad range of psychopathology. Two second-order scales reflecting internalizing and externalizing problems and a total problem score can be calculated. Internal consistency in the present sample was α = 0.96 for the total score, α = 0.85 for the internalizing score, and α = 0.80 for the externalizing score.

The Sibling Questionnaire is a self-developed questionnaire, designed for siblings of adolescents with NSSI and consisting of 166 items [38]. Questions with good face validity were gathered and reviewed by experts. The first part contains demographic questions and asks when siblings first noticed their sister’s NSSI, and if they were told about it, who told them. Further questions refer to the siblings’ suspicions about the reasons for their sister’s self-injury (α = 0.84), questions about the functions of NSSI were formulated on the basis of the Functional Assessment of Self-Mutilation [39] and the Modified Ottawa/Ulm Self-Injury inventory [40]. The second part assesses the siblings’ own experiences with NSSI. In the third part, siblings are asked about their feelings (α = 0.76) and reactions (α = 0.63) when their sister engages in NSSI. The fourth part assesses the impact of NSSI on family dynamics (α = 0.82). Reasons for NSSI, siblings reactions and the impact of NSSI on family dynamics were assessed on a scale ranging from 1 (*fully applies*) to 5 (*does not apply at all*). For siblings feelings, response choices ranged from 1 (*never*) to 5 (*almost always*). Internal consistencies refer to the present sample. So far, the questionnaire has not been further validated.

The Adult Sibling Relationship Questionnaire (ASRQ) [41] measures qualitative features of the sibling relationship in young adulthood and consists of 81 items spread over 14 subscales. The three higher order factors are warmth/closeness, conflict, and rivalry. The warmth subscale consists of items measuring affection, companionship, intimacy, and admiration and the conflict subscale includes quarreling and antagonism between siblings. The rivalry subscale determines whether the parents favor a child, but not which child is favored. All items except rivalry are assessed on a 5-point Likert scale ranging from 1 (*hardly at all*) to 5 (*extremely much*). For the rivalry subscale, response choices are 0 (*neither of us is favored*), 1 (*I am/my sibling is sometimes favored*), and 2 (*I am/my sibling is usually favored*). The questionnaire showed good internal consistency [41]. In the present sample, internal consistency was α = 0.93 for warmth, α = 0.83 for conflict, and α = 0.83 for rivalry.

The Brother–Sister Questionnaire (BSQ) [42] consists of 35 items and is used to distinguish dysfunctional from well-functioning sibling relationships. The BSQ measures the four dimensions empathy (emotional connectedness, caring), boundary maintenance (respect for siblings’ physical and psychological space), similarity (common interests and experiences), and coercion (power and control of one sibling over another). The questionnaire demonstrated good psychometric properties [42]. Internal consistency in the present sample was α = 0.95 for empathy, α = 0.83 for boundary maintenance, α = 0.68 for similarity, and α = 0.52 for coercion.

### Procedure

Participants from the NSSI and CC sample were recruited from nine collaborating child and adolescent psychiatric inpatient clinics. The inpatient clinics were instructed to inform the participants at admission about the study and asked for their consent to participate. Participants from the HC sample were recruited in different high schools. Prior to our visit in the schools, teachers were given detailed information about the study and handed out written informed consent forms, to be signed by the parents of the students participating. After obtaining written informed consent from the adolescents and caregivers, clinical interviews and self-report questionnaires were performed in the inpatient clinics for the NSSI and CC sample and in a classroom after school for the HC group. After data collection for the participants was completed, they were given consent forms and questionnaires for their siblings in case they were willing to participate in the study. Consent form and questionnaires from the siblings were then returned via mail. All participants, adolescents, their siblings and parents, were informed about the study and gave their written consent in accordance with the Declaration of Helsinki. The local ethics committee approved the study.

### Data analyses

We used multivariate analysis of variance (MANOVA) to investigate group differences in sibling relationship. Post hoc tests were conducted to analyze pairwise comparisons. The Bonferroni correction was used to control for multiple comparisons. Effect sizes (Cohen’s *d*) were calculated to further analyze significant group differences. Pearson product-moment correlation coefficients were calculated to evaluate sibling agreement and associations between sibling relationship quality and psychopathology. To compare correlations of sibling agreement, the coefficients were converted to *z* scores. In order to examine adolescent-sibling discrepancies, raw and standardized difference scores were calculated. The standardized difference scores were calculated by subtracting the sibling’s standardized score from the youth’s standardized score [43]. The magnitude of discrepancy between standardized scores was examined by calculating the mean of the absolute value of the difference between standardized scores. All analyses were performed using SPSS version 25. Significance levels were set at α = 0.05.

## Results

### Siblings’ reactions to their sister’s NSSI

Siblings suspected the following reasons for their sister’s self-injury: to change the emotional pain into something physical (60.0%), to relieve tension (57.1%), to deal with frustration (45.0%), and to cope with uncomfortable memories (42.9%). About half of the siblings (57.1%) noticed their sister’s NSSI and the majority (90.5%) were concerned about the behavior. A large proportion (85.7%) believed that their sister might attempt suicide and reported being relieved that their sister was hospitalized. The most common emotional reactions to NSSI were feeling sad (76.2%), depressed (66.7%), desperate (57.1%), helpless (57.1%), angry (33.4%), scared (19.1%), and guilty (14.3%). Several siblings endorsed that they sympathized with their sister (61.9%) and felt distressed due to NSSI (42.9%).

From the perspective of many siblings, the sister’s issues determined the whole family life (42.9%) and they perceived the family situation as very distressing (42.9%). Around a quarter thought that their parents had found a good way to handle their sister’s NSSI (28.6%). Another quarter (23.8%) reported that they did not get their parents’ attention as often as their sisters did and shared the opinion that their parents did not dare to put limits on their sister (23.8%). A third (33.3%) reported supporting their sister by talking with them about NSSI. However, they perceived the conversations as helpful for their sisters (28.6%), but stressful for themselves and indicated that they would like to get help to better cope with their sisters NSSI (28.6%). Many siblings endorsed that they would never understand why their sister is engaging in NSSI (38.1%) and a sizeable proportion felt left alone with the sister’s issues (71.4%). Less than half of the siblings (38.1%) reported being reasonably involved in their sister’s therapy. Those siblings without their own NSSI experience (85.7%) provided several reasons why they did not engage in NSSI (see Table 1). Siblings reported having fewer friends who engage in NSSI (14.3%) than their sister reported for herself (47.6%). Siblings of adolescents with NSSI who also engaged in NSSI (14.3%) were all older siblings who indicated that they had started self-injuring earlier than their sister.

**Table 1:** Siblings of adolescents with NSSI and their reasons for why they not engage in self-injurious behavior (n = 18)

Reason	Number of siblings	%
I have better strategies to deal with stress	9	42.9
I have learned to be thick skinned	9	42.9
I feel less burdened by the family situation	8	38.1
I can express and vent my anger	8	38.1
I have better peer relationships	7	33.4
My sister has experienced more bad things	6	28.6
My sister is too sensitive	5	23.8
I am better at solving problems with our parents	5	23.8
My sister feels more burdened by conflicts with our parents	5	23.8

### Sibling relationship quality

#### Group comparisons based on reports of adolescents with NSSI

Results of the MANOVA showed a significant group difference for the ASRQ subscales warmth, *F*(2, 134) = 7.42, *p *< 0.01, and rivalry, *F*(2, 134) = 14.27, *p *< 0.01. Bonferroni-corrected post hoc analysis revealed that adolescents with NSSI reported significantly less warmth (*p* < 0.01, *d* = 0.73) and more rivalry (*p* < 0.01, *d* = 1.05) in the sibling relationship than NC adolescents. The higher rivalry score indicates parental favoritism for one child by parents of adolescents with NSSI. No difference between groups (NSSI, CC, NC) was found for the ASRQ subscale conflict (see Table 2). Regarding the BSQ subscales the three groups differed significantly on the subscales empathy, similarity, and boundary maintenance. Post hoc analysis showed that adolescents with NSSI reported significantly less empathy (*p* < 0.01, *d* = 0.68) and similarity (*p* < 0.01, *d* = 0.78) than NC adolescents. Adolescents with NSSI reached higher scores in boundary maintenance than NC adolescents (*p* < 0.05, *d* = 0.43), higher scores reflect less concern with boundary maintenance. As shown in Table 2, no group difference emerged for the subscale coercion.

**Table 2:** Means (and standard deviations) derived from the ASRQ and BSQ on sibling relationship quality and the YSR on psychopathological symptoms

Measure	Adolescents					Siblings				
	NSSI (*n *= 56) *M (SD)*	CC (*n* = 33) *M (SD)*	NC (*n* = 50) *M (SD)*	*F*(2, 134)	η^2^	NSSI (*n* = 21) *M (SD)*	CC (*n* = 11) *M (SD)*	NC (*n* = 41) *M (SD)*	*F*(2, 65) *M (SD)*	η^2^
ASRQ										
Warmth	141.27 (38.12)	152.75 (26.82)	164.97 (25.69)	7.42**	0.10	155.62 (21.72)	159.15 (31.88)	154.00 (28.50)	0.14	0.00
Conflict	57.81 (14.49)	57.88 (14.46)	56.64 (13.20)	0.12	0.00	56.11 (13.36)	51.78 (9.51)	55.28 (14.20)	0.33	0.01
Rivalry	0.53 (0.47)	0.38 (0.41)	0.14 (0.23)	14.27**	0.18	0.26 (0.47)	0.10 (0.20)	0.31 (0.44)	0.91	0.03
BSQ										
Empathy	3.12 (0.99)	3.39 (0.88)	3.70 (0.70)	5.39**	0.08	3.36 (0.56)	3.44 (0.70)	3.36 (0.67)	0.06	0.00
Boundaries	4.23 (0.71)	4.20 (0.67)	3.89 (0.88)	3.47*	0.05	2.25 (0.86)	1.89 (0.68)	2.06 (0.59)	0.96	0.03
Similarity	2.41 (0.67)	2.55 (0.46)	2.87 (0.50)	9.29**	0.12	2.78 (0.42)	2.57 (0.39)	2.61 (0.57)	0.83	0.03
Coercion	1.95 (0.68)	1.75 (0.68)	1.71 (0.63)	1.11	0.02	2.11 (0.59)	1.57 (0.19)	1.75 (0.52)	4.43*	0.12
YSR										
INT	35.33 (10.14)	21.70 (9.29)	8.95 (5.87)	120.76**	0.64	10.13 (7.49)	9.04 (6.84)	10.53 (7.92)	0.14	0.00
EXT	19.94 (10.54)	11.61 (6.43)	9.33 (5.39)	23.42**	0.26	9.18 (5.03)	7.60 (5.14)	9.18 (5.03)	0.73	0.02

*NSSI* adolescents with nonsuicidal self-injury, *CC* clinical control group, *NC* nonclinical control group, *ASRQ* Adult Sibling Relationship Questionnaire, *BSQ* Brother Sister Questionnaire, *YSR* Youth Self Report, *INT* Internalizing symptoms, *EXT* externalizing symptoms

* *p *< 0.05

** *p* < 0.01

#### Group comparisons based on siblings’ reports

The only significant difference emerged on the BSQ subscale coercion, *F*(2, 65) = 4.43, *p *= 0.016, η^2^ = 0.12, with post hoc analysis showing that siblings of adolescents with NSSI reported significantly more coercion than CC siblings (*p* < 0.05, *d *= 1.08) and NC siblings (*p* < 0.05, *d *= 0.67); see Table 2. No significant differences were found for the remaining BSQ subscales or any ASRQ subscale.

#### Comparisons between adolescents and siblings in the NSSI group

Significant differences in reports on relationship quality of adolescents with NSSI and their siblings emerged for similarity, *F*(1, 68) = 6.3, *p* < 0.05, η^2^ = 0.09, and boundary maintenance, *F*(1, 68) = 81.07, *p *< 0.01, η^2^ = 0.54, with adolescents with NSSI reporting lower scores on the similarity scale and higher scores on the boundary maintenance scale, indicating less concern with boundary maintenance than their siblings.

### Sibling agreement

The results of sibling agreement are displayed in Table 3. The level of sibling agreement in the NSSI and NC group was low, *r* = 0.05 to 0.35. Siblings of the CC group showed a significant agreement regarding warmth (*r* = 0.74) and similarity (*r* = 0.82). The agreement for both subscales was significantly higher among siblings of the CC group than among NSSI and NC siblings; see Table 3.

**Table 3:** Sibling agreement on dimensions of relationship quality (Pearson correlations)

Measure	NSSI (*n* = 42)	CC n = 22 (*n* = 22)	NC n = 82 (*n* = 82)	*z* scores		
				NSSI vs. CC	NSSI vs. NC	CC vs. NC
ASRQ						
Warmth	0.07	0.74*	0.19	− 2.07*	− 0.43	1.95*
Conflict	0.13	0.52	0.31	− 1.05	− 0.66	0.66
Rivalry	0.11	− 0.03	0.08	0.33	0.11	− 0.28
BSQ						
Empathy	0.12	0.42	0.31	− 0.77	− 0.70	0.33
Boundaries	0.20	0.58	0.06	− 1.08	0.50	1.55
Similarity	0.35	0.82*	0.25	− 1.86*	0.39	2.32*
Coercion	0.05	0.29	− 0.24	− 0.56	1.03	1.40

*NSSI* adolescents with nonsuicidal self-injury, *CC* clinical control group, *NC* nonclinical control group, *ASRQ* Adult Sibling Relationship Questionnaire, *BSQ* Brother–Sister Questionnaire

* *p *< 0.05

** *p* < 0.01

In addition to sibling agreement, Table 4 reflects sibling discrepancies showing raw and standardized difference scores as well as absolute value standardized differences. There was considerable variability among the difference scores, as indicated by large standard deviations of the raw discrepancy. The mean of the absolute value of the difference between standard scores indicates that the difference between adolescent and sibling reports in the CC and NC group was small for most aspects of relationship quality with less than one standard deviation (< 1). The NSSI group showed the largest discrepancies (> 1 for most subscales).

**Table 4:** Raw, standardized and absolute value standardized differences scores for adolescent and sibling reports of sibling relationship quality

Measure	Difference scores								
	Raw *M* (*SD*)			Standardized *M* (*SD*)			Absolute Value Standardized *M* (*SD*)		
	NSSI (*n* = 42)	CC (*n* = 22)	NC (*n* = 82)	NSSI (*n* = 42)	CC (*n* = 22)	NC (*n* = 82)	NSSI (*n* = 42)	CC (*n* = 22)	NC (*n* = 82)
ASRQ									
Warmth	3.11 (37.52)	− 6.08 (23.15)	9.50 (34.40)	− 0.08 (1.36)	− 0.41 (0.84)	0.16 (1.24)	1.09 (0.76)	0.74 (0.54)	0.86 (0.90)
Conflict	− 1.79 (19.90)	5.20 (11.68)	1.88 (15.68)	− 0.28 (1.52)	0.25 (0.89)	− 0.00 (1.20)	1.11 (1.04)	0.74 (0.48)	0.92 (0.76)
Rivalry	0.27 (0.48)	0.20 (0.42)	− 0.16 (0.49)	0.76 (1.36)	0.51 (1.15)	− 0.40 (1.17)	1.15 (0.99)	0.75 (0.97)	0.87 (0.90)
BSQ									
Empathy	0.36 (0.96)	0.12 (0.86)	0.30 (0.81)	0.11 (1.37)	− 0.22 (1.25)	0.04 (1.18)	1.11 (0.76)	0.89 (0.85)	0.76 (0.90)
Boundaries	1.88 (1.23)	2.24 (0.73)	1.84 (0.95)	− 0.16 (1.70)	0.40 (0.99)	− 0.15 (1.28)	1.32 (1.03)	0.81 (0.63)	0.98 (0.83)
Similarity	− 0.08 (0.64)	0.07 (0.32)	0.21 (0.64)	− 0.27 (1.18)	− 0.02 (0.60)	0.26 (1.17)	0.93 (0.76)	0.50 (0.28)	0.91 (0.76)
Coercion	− 0.25 (1.05)	0.07 (0.32)	− 0.05 (0.83)	− 0.38 (1.82)	0.32 (0.54)	0.08 (1.48)	1.37 (1.23)	0.49 (0.36)	1.11 (0.95)

*NSSI* adolescents with nonsuicidal self-injury, *CC* clinical control group, *NC* nonclinical control group, *ASRQ* Adult Sibling Relationship Questionnaire, *BSQ* Brother–Sister Questionnaire

### Association between sibling relationship quality and psychopathology in the NSSI and CC group

Correlations between sibling relationship quality and psychopathology are presented separately for adolescents with NSSI and their siblings in Tables 5 and 6. Among adolescents with NSSI a significant association was found between internalizing problems and coercion as well as externalizing problems and similarity (both *r *= 0.27). For adolescents in the CC group significant associations emerged between internalizing problems and conflict (*r* = 0.35) and boundary maintenance (r = − 0.47) as well as externalizing problems and conflict (*r *= 0.47), similarity (*r* = 0.37) and coercion (*r *= 0.35). In the NSSI group siblings’ reports showed that internalizing problems were significantly associated with warmth, conflict, and empathy (all *r* = 0.48) in the sibling relationship. No associations between sibling relationship quality and psychopathology were found in reports of siblings in the CC group. Siblings of the three groups did not differ significantly regarding internalizing, *F*(2, 65) = 0.14, *p* > 0.05, or externalizing, *F*(2, 65) = 0.73, *p* > 0.05, problems.

**Table 5:** Pearson correlations of sibling relationship quality (ASRQ, BSQ) and psychopathological symptoms (YSR) reported by adolescents with nonsuicidal self-injury disorder

Measure	1	2	3	4	5	6	7	8
ASRQ								
1. Warmth	–							
2. Conflict	− 0.54**	–						
3. Rivalry	− 0.33*	0.15	–					
BSQ								
4. Empathy	0.88**	− 0.61**	− 0.30*	–				
5. Boundaries	0.03	− 0.36**	− 0.09	0.20	–			
6. Similarity	0.62**	− 0.31*	− 0.35*	0.63**	0.16	–		
7. Coercion	− 0.42**	0.50**	0.23	− 0.50**	− 0.22	− 0.17	–	
YSR								
8. Internalizing problems	− 0.03	0.11	0.13	− 0.01	0.11	− 0.04	0.27*	–
9. Externalizing problems	0.11	0.02	0.22	0.19	0.15	0.27*	0.18	0.38**

*ASRQ* Adult Sibling Relationship Questionnaire, *BSQ* Brother–Sister Questionnaire, *YSR* Youth Self-Report

* *p *< 0.05

** *p* < 0.01

**Table 6:** Pearson correlations of sibling relationship quality (ASRQ, BSQ) and psychopathological symptoms (YSR) reported by siblings of adolescents with nonsuicidal self-injury disorder

Measure	1	2	3	4	5	6	7	8
ASRQ								
1. Warmth	–							
2. Conflict	0.02	–						
3. Rivalry	− 0.33	0.06	–					
BSQ								
4. Empathy	0.72**	0.08	− 0.11	–				
5. Boundaries	− 0.20	0.54*	0.20	0.19	–			
6. Similarity	0.12	0.07	− 0.04	0.15	0.19	–		
7. Coercion	− 0.20	0.60**	0.11	0.12	0.76**	0.01	–	
YSR								
8. Internalizing problems	0.48*	0.48*	− 0.01	0.48*	0.35	− 0.13	0.18	–
9. Externalizing problems	0.32	0.13	− 0.29	0.28	0.02	0.33	0.02	0.37

*ASRQ* Adult Sibling Relationship Questionnaire, *BSQ* Brother–Sister Questionnaire, *YSR* Youth Self-Report

* *p *< 0.05

** *p* < 0.01

## Discussion

This study is the first to address siblings’ reactions to a sister’s NSSI as well as aspects of sibling relationship quality, such as warmth, rivalry, coercion, and conflict, group differences (adolescents with NSSI, CC, NC) with respect to sibling relationship quality, agreement between adolescents with NSSI, CC, and NC and their siblings, and the association between sibling relationship quality and psychopathology separately for adolescents with NSSI and their siblings.

Consistent with previous research on parental reports of siblings’ emotional reactions to NSSI [21, 22], siblings involved in this study described their sister’s NSSI as being a source of distress, sadness, desperation, helplessness, and anger. The majority of siblings was concerned about their sister’s NSSI as well as potential future suicidal behavior and felt relieved about their sister receiving inpatient psychiatric treatment. A third of siblings supported their sister by talking to her about NSSI and although they considered these conversations helpful for their sister, they perceived them as distressing for themselves and wished for help to cope better with NSSI. In fact, 71.4% of siblings felt left alone with the sister’s issues and 38.1% will never understood why their sister was engaging in NSSI. These findings highlight the need to provide sufficient psychoeducation for family members to increase their understanding of the behavior and enhance the family’s communication and coping skills [44]. Relatives of individuals with a mental disorder have been shown to benefit from psychoeducational support groups [45, 46]. Based on the siblings’ reports in our study, NSSI has a negative impact on emotional well-being and family life, which raises the question of whether these siblings might be at risk of developing their own mental health issues. Research on siblings of individuals with mental disorders has reported high levels of emotional distress, especially if the sibling is still living with the family [47]. However, we found no group differences between siblings in the three groups with respect to internalizing or externalizing symptoms. Nonetheless, given the reported emotional impact of NSSI, the feeling of being left alone with their sister’s issues, and the wish for support, it is crucial to create opportunities for siblings to address their worries about NSSI and to receive support. Due to their extensive contact during childhood and adolescence, siblings are often key family members and can be a great source of emotional and practical support [48, 49]. Siblings can help promote the well-being and recovery of a sibling with a mental disorder, through engaging jointly in appropriate activities, for example, exercise or sports, or integrating the sibling in their social circle [50].

Adolescents with NSSI reported significantly less warmth, empathy, and similarity and more rivalry in the sibling relationship than NC adolescents. Furthermore, they indicated significantly less concern with boundary maintenance compared to NC adolescents. Adolescents with NSSI felt less emotionally connected with their sibling and reported lower empathy, caring, intimacy, similarity, and companionship in their sibling relationship compared to NC adolescents. There is some research indicating that children and adolescents might have similar experiences with siblings and peers in terms of relationship quality [51–53]. A study by Pike and Atzabe-Poria [51] found that sibling affection predicted greater positivity in their best friendships, while greater sibling hostility was related to lower positivity and greater conflict with friends. Similarly, among children, sibling warmth was positively associated with best friendship quality, whereas sibling conflict was negatively associated with friendship quality [53]. A poorer relationship quality with their siblings might be associated with the peer problems of adolescents with NSSI [26, 54]. Adolescents with NSSI report significantly less perceived social support from friends and family as well as having fewer people to seek advice from than healthy controls, which supports the notion that they experience difficulties with forming relationships and developing adaptive interpersonal skills [26]. In order to deal with these negative emotional states emerging from stressful peer experiences, NSSI may be used as a coping mechanism [55].

Adolescents with NSSI reported significantly higher rivalry scores than NC adolescents, suggesting that parents of adolescents with NSSI favor one child over another more than NC parents do. The rivalry subscale comprises items assessing maternal and paternal favoritism. This finding can be interpreted in light of research emphasizing that the self-injuring child becomes the center of familial attention, leading to an imbalance in parental involvement between siblings [22–25]. Similarly, almost a quarter of siblings of adolescents with NSSI represented in this study experienced less parental attention compared to their sister and believed that their parents were having difficulties setting boundaries. Furthermore, a considerable proportion of siblings endorsed the suggestion that the sister’s issues determined family life for the whole family (42.9%). However, no group differences on the rivalry subscale between siblings emerged, indicating no group differences with respect to parental favoritism from the sibling’s point of view. Differential parental treatment can have a negative impact on family dynamics and sibling relationships and is associated with greater sibling conflict, antagonism, and controlling behaviors [56–58]. The parental favoritism reported in families of adolescents with NSSI might contribute to the maladaptive family functioning, which has been found to contribute to maintaining NSSI [11, 13]. Adolescents with NSSI have significantly greater success in having their boundaries respected by their siblings compared to NC siblings, which might be linked to our finding that the siblings of adolescents with NSSI reported significantly more coercion than both CC and NC adolescents. As adolescents with NSSI showed more dominance and control over their siblings, it might be easier for them to maintain their boundaries.

Siblings of adolescents with NSSI scored significantly higher on the coercion subscale compared to CC and NC siblings, emphasizing the dominance and control of adolescents with NSSI in their sibling relationship. Studies have shown that high levels of psychological control from a sibling is associated with ill-being, adjustment problems, and anxiety and depressive symptoms in the victimized sibling [59–61]. However, coercion was not associated with internalizing and externalizing problems in siblings of adolescents with NSSI. As no clinical cut-off score for the coercion scale exists, it is difficult to determine whether coercion levels in the sibling relationship of adolescents with NSSI are abnormal or not. However, as siblings in the NSSI group scored higher than both CC and NC siblings, this issue requires further elaboration in future studies.

Our results showed that siblings of adolescents with NSSI involved in this study scored significantly lower on the boundary maintenance scale of the BSQ than their sisters, reflecting difficulties in establishing and respecting firm and reasonable interpersonal boundaries between siblings [42]. Lower scores indicate that the siblings fail to have their boundaries respected by their sisters with NSSI. Furthermore, adolescents with NSSI scored significantly lower on the similarity subscale than their siblings, indicating that they see themselves as more de-identified and different from their siblings and having less in common compared to their siblings rating. Previous research has shown that NSSI is associated with identity confusion [62] and may provide a source of self-identification [63]. Considering this, it is not surprising that adolescents with NSSI don’t identify themselves with their siblings but see themselves as different.

Overall, sibling agreement in the NSSI group was low, indicating somewhat diverging perceptions of all relationship quality dimensions used in this study. This result differs from an earlier study that found a substantial sibling agreement for the ASRQ subscales warmth, conflict, and rivalry [64]. However, the average age of participants (20.60 years) and siblings (23.00 years) was higher than the average age of participants (16.18 years) and siblings (16.88) in this study. Although adolescent and sibling reports in this study differed for most aspects of sibling relationship quality, the magnitude of these discrepancies was quite small, as measured by standardized scores. Adolescents in the CC group showed the best sibling agreement, especially on the subscales warmth and similarity. This result might be explained by differences in the group sizes and should be further examined with larger CC samples.

Dimensions of sibling relationship quality were only moderately associated with psychopathological symptoms among both adolescents with NSSI and their siblings. Among adolescents with NSSI externalizing problems were significantly associated with similarity in the sibling relationship, whereas internalizing problems were significantly associated with coercion.

The first mentioned association can be interpreted in line with previous research showing that high levels of intimacy (as a proxy for similarity) among siblings close in age might increase the affective intensity of their conflicts [65, 66], thereby leading to higher levels of aggression. Coercion in sibling relationships can be seen as important learning experience, since siblings influence each other’s aversive and aggressive behavior, e.g., through reinforcement [67]. However, behavioral changes resulting from hostile sibling interactions can cause internalizing symptoms [68].

Among siblings of adolescents with NSSI internalizing problems were significantly associated with conflict, warmth, and empathy. The association between conflict and internalizing problems is consistent with previous research showing that greater sibling conflict during childhood and adolescence leads to higher internalizing symptoms [16], especially when siblings are close in age [57]. The association between high levels of warmth and empathy and internalizing problems may indicate that in close sibling relationships, the sisters mental health issues and NSSI might lead to worries and a negative emotional impact on their sibling, resulting in elevated levels of internalizing symptoms. For adolescent friendships, co-rumination, excessive discussion of interpersonal problems, and negative feelings were found to be associated with high-quality friendships but also with greater internalizing symptoms [69]. This may also count for close siblings of adolescents with NSSI, who spend much time discussing their sister’s problems.

In light of our finding that the relationship between adolescents with NSSI and their siblings is characterized by less warmth, empathy, and similarity and more coercion than in the NC group, and the well established link between poor sibling relationship quality and emotional and behavioral problems, indicates that sibling interventions (in terms of increasing warmth and reducing conflict) might be beneficial in reducing psychopathological symptoms, for a review see Dirks et al. [17]. However, promoting more engaged and positive sibling relationships may in turn yield the danger of increasing the emotional distress of the sibling, as outlined above. A review on susceptibility to environmental influences highlights that some characteristics such as genetic or temperament factors may leave an individual more resistant or prone to both negative and positive environmental influences [70]. Thus, some children and adolescents might perceive negative sibling experiences as more distressing than others, or might be more likely to benefit from promoting positive sibling interactions [17]. Future research is necessary to determine the circumstances in which incorporating treatment components targeting sibling relationships or family dynamics may be beneficial for improving psychological symptoms [17].

Despite the fact that sibling conflicts and aggression can have severe negative consequences for children’s and adolescent’s well-being, we only have a very limited understanding of evidence-based programs promoting positive sibling relationships. Preliminary evidence for the improvement of sibling relationship quality among school-aged children has been found for interventions targeting children’s social skills (for a review see [71]). These interventions either directly improve social skills in sibling interactions via trained professionals or indirectly by focusing on training parents on mediation skills. However, more research is needed with respect to interventions preventing or intervening with sibling conflict and aggression.

The results of the present study should be interpreted in the context of the following limitations. The sample consisted of female adolescents admitted to an inpatient child and adolescent psychiatric unit and thus may not generalize to other samples or to male adolescents. The design of the study was cross-sectional. Therefore, the current study cannot explain the direction of effects between an adolescent’s NSSI and sibling relationship quality and family dynamics. This should be investigated in future prospective longitudinal studies and on the basis of a larger sample size, including male and female adolescents. Boys who self-injure are a quite understudied population. The literature indicates that boys and girls differ with respect to basic NSSI characteristics such as methods, location, and functions, supporting the idea that interventions should be gender-specific. Given that male-preferred methods of NSSI include hitting and burning, the nature of the behavior might be perceived as aggressive rather than self-injurious, thereby masking the true intention [72]. In light of these differences, it is possible that NSSI performed by boys might elicit a different response from parents and siblings compared to a self-injuring girl, however future studies on this matter are needed. To date, there is not sufficient data to answer the question whether brothers might have a different coping of their sisters NSSI than a female sibling. Studies in children and adolescents suggest that gender composition and age difference of sibling pairs have a moderating effect on sibling relationship quality, which in turn might influence how siblings cope with maladjustment [16]. Thus, it is possible that a brother copes differently with his sisters NSSI than with a brothers NSSI and vice versa. Further, adolescents with NSSI may perceive their sibling relationship as less warm and supportive due to a negative cognitive bias, this should be addressed in future studies. More research into rivalry is needed in order to understand, which child is favored by parents of adolescents with NSSI and to investigate sibling rivalry, since this study only considered parental rivalry. Another, unavoidable limitation was the use of a non-validated questionnaire for the assessment of sibling relationship quality. Nevertheless, we addressed a neglected research question. Strengths of the study were the use of the *DSM*-*5* diagnostic research criteria for NSSI and the use of a multi-informant approach, including adolescent and sibling reports as well as the inclusion of a clinical and a nonclinical control group.

## Conclusions

Adolescents with NSSI differed significantly with respect to many dimensions of sibling relationship quality compared to the non-clinical controls (NC), but not compared to the clinical controls (CC). We found that the CC group did not differ from adolescents with NSSI nor to the NC group, indicating that differences between the NSSI and the NC group may be attributed to a characteristic of the NSSI group. However, more research is required to explore this relationship in further detail. We found significant differences between all three groups regarding the BSQ subscale coercion, emphasizing the dominance, and control of adolescents with NSSI in their sibling relationship compared to both the CC and NC group. Similarly, our results indicate that siblings fail to have their boundaries respected by their sisters with NSSI. Despite the fact that we found differences only between adolescents with NSSI and NC, significant differences between all three groups were found among siblings, indicating a NSSI-specific association. Since this manuscript aims to highlight the impact of NSSI on siblings and the siblings’ view on sibling relationship quality, we believe that this manuscript adds important findings to the literature.

According to the siblings represented in our study, NSSI is associated with poor emotional well-being and family life, as the family attention frequently centers on concerns related to the sister’s mental health issues. These results underline the importance of a sibling support component for siblings of adolescents with NSSI to help them cope with the emotional and familial consequences of their sister’s NSSI and to prevent and reduce any negative emotional impact in the long term.

## Abbreviations

NSSI: nonsuicidal self-injury
CC: clinical controls
NC: nonclinical controls
ASRQ: Adult Sibling Relationship Questionnaire
BSQ: Brother–Sister Questionnaire
DSM: Diagnostic and Statistical Manual of Mental Disorders
Kinder-DIPS: Diagnostic Interview for Mental Disorders in Children and Adolescents
SCID-II: Structured Clinical Interview for *DSM*-*IV* Axis II disorders
YSR: Youth Self-Report
MANOVA: multivariate analysis of variance
